# Marathon running pace immediately before sudden cardiac arrest

**DOI:** 10.1016/j.ajpc.2025.101390

**Published:** 2025-12-21

**Authors:** Jo Kato, Tomohiro Manabe, Fumihiro Yamasawa

**Affiliations:** a Department of Cardiology, NHO Kasumigaura Medical Center, Tsuchiura, Japan; b Medical Committee, Japan Association of Athletics Federations (JAAF), Tokyo, Japan; c Sports Medicine Research Center, Keio University, Yokohama, Japan; d Marubeni Health Promotion Center, Tokyo, Japan

**Keywords:** Sudden cardiac arrest, Full marathon, Running pace

## Abstract

**Background:**

Sudden cardiac arrest (SCA) is a rare but catastrophic event that can occur during long-distance road races. Although habitual training mitigates SCA risk, it remains uncertain whether running pace on race day can help identify susceptible individuals.

**Methods:**

We prospectively collected cases of SCA in Japan Association of Athletics Federations (JAAF)-certified full marathons between April 2011 and March 2020. Collapses during or within 1 hour after races that required basic life support were included. Running pace was calculated from the last available split or finish time, and expected completion times were compared with age- and sex-stratified marathon ranking data. Predicted finish time percentiles were evaluated within subgroups defined by calendar year, sex, age group, and location of collapse (race tertile or postfinish).

**Results:**

Among 4.53 million starters in 571 marathons, 74 SCA cases were identified (1.6/100,000). The median age was 52 years, and 93% were men. Over half of the events occurred in the final tertile or immediately postfinish. The median pace was 10 minutes 25 seconds per mile (interquartile range: 9:15–12:13), with an extrapolated finish time of 4 hours 33 minutes, corresponding to the 48th percentile in population rankings. Females and those collapsing in the latter part of the race tended to occupy higher percentile ranks than the general finisher distribution.

**Conclusions:**

Marathon-related SCA occurred at running speeds indistinguishable from the general finisher population, challenging the assumption that less conditioned runners are particularly at risk of SCA.

## 1 Introduction

Sudden cardiac arrest (SCA) during sporting activities occurs in approximately 1 out of 50,000 to 80,000 person-years [1]. In mass-participation marathons, the same absolute risk is compressed into a single race day: with approximately 1 SCA occurring in every 60,000 to 100,000 runners [[2], [3], [4], [5]]. A single participation in a full marathon represents an equivalent SCA risk of 1 year of other sporting activities, thereby providing a unique opportunity to capture this extremely rare event.

Understanding the characteristics of SCA victims is essential for developing preventative strategies and optimizing on-course emergency preparedness during long-distance road races. Although habitual exercise may blunt the cardiovascular stress of vigorous exertion [6], it remains unclear whether the pace sustained on race days can be used to identify individuals who will collapse. To address this question, we analyzed the pre-collapse pace profiles of runners who experienced SCA during the Japan Association of Athletics Federations (JAAF)-certified marathons.

## 2 Methods

### 2.1 Study design

This was a prospective observational study aggregating cases of SCA in JAAF-certified full marathons [2]. This study was conducted in accordance with the Strengthening the Reporting of Observational Studies in Epidemiology (STROBE) guidelines. The Ethics Review Committee of the Sports Medicine Research Center at Keio University approved this study (No. 2013–03, April 1, 2013) and waived the requirement for written informed consent, because no individually identifiable data were collected.

### 2.2 Study population

We prospectively recorded cases of SCA in JAAF-certified marathons held between April 2011 and March 2020. After each race, a questionnaire was sent to the race directors. Local newspaper archives and web reports were searched simultaneously for information. Discrepancies were resolved through direct contact with the race office. SCA was defined as a collapse occurring during the race or within 1 hour after finishing, followed by basic life support (BLS), including chest compressions and/or defibrillation.

### 2.3 Pace calculation

For each case, we calculated the running speed (distance per elapsed time) and pace (time per unit distance). If the collapse occurred after completion, the official finish time was used. Assuming that the observed pace was maintained over the full marathon distance (26.2 miles), the expected finish time was extrapolated from the data. Annual, age-, and sex-stratified finisher tables for more than 30 marathons were obtained from RUNNET [7]. Each estimated finish time was assigned a percentile rank within the appropriate stratum for that year. As supplemental reference data, the average finish time and corresponding 95 % confidence interval for each stratum were also extracted. The analyses were stratified by calendar year, sex, age group (<40, 40–49, 50–59, and ≥60 years), and location of collapse (tertile of race distance, or postfinish). For the sensitivity analysis, we applied the same calculations to the half-marathon SCA cases reported to the JAAF during the same period.

### 2.4 Statistical analyses

Continuous variables were summarized as medians with interquartile ranges (IQRs), whereas marathon ranking data were presented as means with 95 % confidence intervals according to their distribution. The incidence of SCA was calculated as the number of cases per 100,000 starters overall and within subgroups stratified by age, sex, calendar period, and race distance at collapse. The denominators for age-group–specific incidence were calculated according to the method described in reference 2. Comparisons of percentile ranks between subcategories were performed using the Wilcoxon rank-sum test, with Bonferroni correction applied for multiple comparisons. Incidence comparisons were performed using Poisson regression. All analyses were performed using R software (version 4.5.0; R Foundation for Statistical Computing, Vienna, Austria). A two-sided *P* value <0.05 was considered statistically significant.

## 3 Results

During the 10 years, 571 certified marathons attracted 4.53 million starters. Seventy-four SCA events were documented across 61 races (overall incidence: 1.6/100,000). The median patient age was 52 years (interquartile range [IQR]: 44–61 years), and 69 (93.2 %) cases occurred in men. Among 55 runners with documented timepoints, the median interval from collapse to initiation of BLS was 1 min (IQR: 0.5–2); spontaneous circulation was restored in 73 of 74 patients (98.6 %). The site and timestamp of collapse were available for 71 patients (67 men and 4 women). More than half of the cases (56.1 %) occurred in the final tertile of the course or immediately after completion.

The overall median speed was 5.8 miles/h (IQR: 4.9–6.5), corresponding to a median pace of 10 min 25 s per mile (IQR: 9:15–12:13) (Table 1). The observed speeds ranged from 1.4 miles/h to 11.3 miles/h, equivalent to paces of 42 min 05 s to 5 min 17 s per mile. The extrapolated finish time was 4 h 32 min 52 s (IQR: 4:02:37–5:19:59), which closely matched the population mean in the marathon ranking data. Within these distributions, the median percentile rank of SCA cases was 48 % (IQR: 26–75).

**Table 1:** Median running speed, pace, predicted completion time, and percentile rank among runners with sudden cardiac arrest during full marathon events, stratified by incidence year, sex, age group, and sudden cardiac arrest occurrence distance.

	Number of SCA	Running speed,	Running pace,	Predicted completion time,	Marathon ranking time,^a^	Percentile rank,	Incidence of SCA b
		miles per hour,	min:s per mile,	h:min:ss,	h:min:ss,		per 100,000 runners
		median (IQR)	median (IQR)	median (IQR)	mean (95 % CI)	median (IQR)	(n/N)
Overall	71	5.8	10:25	4:32:52	4:36:19	48	1.6
		(4.9–6.5)	(9:15–12:13)	(4:02:37–5:19:59)	(4:36:15–4:36:23)	(26–75)	(74/4528,134)
Incidence year c							
2011–2014	18	5.2	11:34	5:03:08	4:43:47	73	1.4
		(4.7–6.2)	(9:43–12:42)	(4:14:36–5:32:41)	(4:43:39–4:43:55)	(42–86)	(18/1246,793)
2014–2017	28	6.0	10:02	4:23:03	4:43:37	35	1.8
		(5.0–6.7)	(8:58–12:02)	(3:54:55–5:15:48)	(4:43:30–4:43:44)	(23–73)	(29/1654,908)
2017–2020	25	5.8	10:25	4:32:54	4:23:32	45	1.7
		(5.1–6.5)	(9:13–11:46)	(4:01:15–5:08:04)	(4:23:25–4:23:39)	(22–74)	(27/1626,462)
Sex							
Men	67	6.2	9:43	4:14:46	4:28:13	51 d	1.9 e
		(5.3–6.9)	(8:45–11:27)	(3:49:25–5:00:12)	(4:28:09–4:28:18)	(27–76)	(67/3548,582)
Women	4	5.7	10:28	4:34:25	5:07:01	21	0.4
		(4.9–6.5)	(9:16–12:14)	(4:02:58–5:20:45)	(5:06:51–5:07:10)	(7–33)	(4/923,176)
Age groups							
<40s	14	6.1	9:55	4:20:00	4:42:40	30	0.9
		(5.2–7.2)	(8:23–11:32)	(3:39:48–5:02:23)	(4:42:33–4:42:47)	(10–59)	(14/1507,695)
40s	15	6.2	9:43	4:14:46	4:34:12	44	1.0
		(5.3–6.6)	(9:05–11:16)	(3:58:09–4:55:24)	(4:34:06–4:34:19)	(29–72)	(15/1431,456)
50s	24	5.4	11:07	4:51:28	4:31:39	65	3.0 e
		(4.8–6.2)	(9:41–12:26)	(4:13:53–5:25:59)	(4:31:30–4:31:47)	(34–84)	(24/806,517)
≥60s	18	5.4	11:06	4:51:02	4:45:34	53	5.2 e
		(4.8–6.2)	(8:53–12:33)	(3:52:55–5:29:03)	(4:45:20–4:45:48)	(11–80)	(18/345,321)
Site where SCA occurred							
≤8.8 miles	11	4.1	14:49	6:28:29	NA	98 f	NA
		(2.3–5.3)	(11:16–26:26)	(4:55:24–11:33:03)		(68–100)	
8.9–17.5 miles	19	5.4	11:05	4:50:36	NA	59	NA
		(4.9–6.2)	(9:40–12:14)	(4:13:27–5:20:45)		(40–75)	
17.6–26.2 miles	32	6.3	9:32	4:09:57	NA	31	NA
		(5.3–6.7)	(8:58–11:17)	(3:55:06–4:55:50)		(18–57)	
After completion	9	5.8	10:25	4:33:07	NA	48	NA
		(4.9–6.3)	(9:33–12:07)	(4:10:23–5:17:41)		(27–75)	

a Marathon ranking data were derived from the annual registry of approximately 300,000 marathon finishers in Japan. For each year, only the fastest record of each runner (stratified by age and sex) was used to construct the rankings.

b Incidence rates were calculated based on the available data for each subgroup.

c Year categories are based on the Japanese fiscal year (April–March).

d *P* < 0.05 by Wilcoxon test for sex comparison.

e *P* < 0.05 by Poisson regression (vs. female for sex, vs. 40 s for age groups).

f Bonferroni-corrected *P* < 0.008 by the Wilcoxon test for each pair. CI, confidence interval; IQR, interquartile range; NA, not applicable; SCA, sudden cardiac arrest.

The incidence of SCA differed among the subgroups. The incidence remained largely unchanged across the three study periods. Men had a higher incidence than women (1.9 vs 0.4 per 100,000; *P* < 0.05), while runners ≥60 s years exhibited the highest incidence (5.2 per 100,000) compared with those in their <40 s (0.9 per 100,000; *P* < 0.05). Performance percentiles did not differ significantly by age group; however, women achieved higher percentile ranks than men, despite similar absolute running speeds (*P* < 0.05). By site of collapse, cases in the first tertile occurred at significantly slower paces, whereas those in the final tertile or after completion occurred significantly faster and ranked above the population average (Bonferroni-corrected *P* < 0.008).

Fifteen cases of SCA were identified in the half-marathons. Their median speed (5.7 miles/h; IQR: 4.9–6.6) and pace (10 min 32 s per mile; IQR: 9:09–12:23) paralleled the findings from the full marathon.

## 4 Discussion

We identified 74 marathon SCA cases over a decade, with a crude incidence of approximately 1.6 per 100,000 runners. This rate is slightly higher than that reported in the US-based *Race Associated Cardiac Event Registry* (RACER) [3] and European data [5]. However, the difference can be explained primarily by the greater proportion of older participants in Japan. In our cohort, the median age of patients who experienced SCA was 52 years (mean 51.1), whereas a mean age of 42.3 years was found in RACER, suggesting an older at-risk population.

Precollapse pace percentiles, with a median of 48 % and IQR of 26 %–75 %, approximated the general finisher distribution, challenging the assumption that inadequate physical conditioning is a common risk factor for exercise-related SCA. We recognize that the marathon pace is only a proxy, and an imperfect metric, for habitual training. However, completing 26.2 miles in approximately 4.5 h generally requires a sustained endurance exercise program. Given that most runners with late-onset SCA were running at above-average paces, our findings do not support the hypothesis that inadequate physical capacity underlies these events.

Therefore, our findings indicate that SCA risk spans a broad performance spectrum, suggesting that stratification based on self-reported finish time (seed time) is unlikely to be an effective approach. Since 2014, the JAAF Medical Committee has recommended that all participants use a checklist of symptoms and risk factors to encourage runners to assess their cardiovascular risk prior to participating in races [2]. However, as the incidence of SCA had remained unchanged over the study period, even such measures may not contribute to a meaningful reduction in risk. Further refinement of preventive strategies is warranted, either by improving universal self-screening tools or by focusing more specifically on subgroups with high-risk attributes in mass participation events.

Finally, given the inherent limitations of primary prevention, equal emphasis should be placed on the rapid recognition and prompt treatment of SCA to improve survival. The etiology of SCA is heterogeneous, with approximately half of the cases attributed to ischemic heart disease [3,4]; however, the effectiveness of early basic life support is well established, regardless of the underlying etiology. Thus, preparedness for immediate resuscitation remains the cornerstone of reducing mortality in mass-participation marathon events.

The major strength of this study is that it represents the first investigation to systematically evaluate exercise performance profiles in patients with marathon-related SCA, providing novel insights into their precollapse characteristics. The limitations of this study include the potential double-counting of repeat marathoners, the absence of data on prodromes, training history, and cardiovascular risk factors, unknown etiology in each case, and an unavoidable time lag between collapse and event recognition. Additionally, marathon pace is an imperfect surrogate for cardiorespiratory fitness because it can be influenced by factors such as dehydration, environmental conditions (e.g., ambient temperature and wind speed), and subclinical illness. Our pace estimates were based on the last recorded split and could not account for terminal deceleration.

In conclusion, marathon SCA occurred at running speeds indistinguishable from the general finisher population, indicating that the risk is not confined to slower or less-conditioned runners. This finding underscores the need for continued emphasis on universal preparedness and rapid on-site resuscitation, as well as refining strategies for prerace screening.

## Funding

This research did not receive any grants.

## Data availability statement

Data regarding this article will be shared upon reasonable request from the corresponding author.

## CRediT authorship contribution statement

**Jo Kato:** Writing – original draft, Methodology, Formal analysis. **Tomohiro Manabe:** Writing – review & editing, Investigation, Data curation. **Fumihiro Yamasawa:** Writing – review & editing, Supervision, Conceptualization.

## Declaration of competing interest

The authors declare that they have no known competing financial interests or personal relationships that could have appeared to influence the work reported in this paper.
